# Signal transduction around thymic stromal lymphopoietin (TSLP) in atopic asthma

**DOI:** 10.1186/1478-811X-6-5

**Published:** 2008-08-25

**Authors:** Katrin Sebastian, Andreas Borowski, Michael Kuepper, Karlheinz Friedrich

**Affiliations:** 1 Institute of Biochemistry, University of Jena Medical School, Germany; 2 Department of Pneumology, Medical University Clinic, Rostock, Germany

## Abstract

Thymic stromal lymphopoietin (TSLP), a novel interleukin-7-like cytokine, triggers dendritic cell-mediated inflammatory responses ultimately executed by T helper cells of the Th2 subtype. TSLP emerged as a central player in the development of allergic symptoms, especially in the airways, and is a prime regulatory cytokine at the interface of virus- or antigen-exposed epithelial cells and dendritic cells (DCs). DCs activated by epithelium-derived TSLP can promote naïve CD4+ T cells to adopt a Th2 phenotype, which in turn recruite eosinophilic and basophilic granulocytes as well as mast cells into the airway mucosa. These different cells secrete inflammatory cytokines and chemokines operative in inducing an allergic inflammation and atopic asthma. TSLP is, thus, involved in the control of both an innate and an adaptive immune response. Since TSLP links contact of allergen with the airway epithelium to the onset and maintainance of the asthmatic syndrome, defining the signal transduction underlying TSLP expression and function is of profound interest for a better understandimg of the disease and for the development of new therapeutics.

## Background

Atopic asthma is a common inflammatory disorder of the airway epithelium characterized by tissue obstruction and remodeling, bronchial smooth muscle cell hyperreactivity to allergens and chronic bronchial inflammation. It classically involves allergen-driven T helper 2 (Th2) lymphocyte polarisation with coordinate production of interleukin (IL)-4, IL-5, IL-13 and granulocyte-macrophage colony-stimulating factor (GM-CSF), which are encoded in one gene cluster on chromosome 5q31-34 [1]. IL-4 and IL-13 are critically involved in the pathogenesis of allergic asthma by regulating IgE-production by B cells, inducing airway hyperreactivity and triggering key features of airway remodeling, whereas IL-5 is a key factor for eosinophilia [2,3]. Activation of IgE receptors on mast cells triggers the release of preformed vasoactive mediators such as histamine, the synthesis of prostaglandins and leukotrienes, and, via a positive feedback loop, expression IL-4 and IL-13 [2].

Its apparent association with airway diseases has recently focussed interest on the novel IL-7-like cytokine thymic stromal lymphopoietin (TSLP). TSLP expression is increased in asthmatic airways and correlates with both the expression of Th2-attracting chemokines and with disease severity [4-6], indicating a link between TSLP and human asthma. Furthermore it was shown that experimental lung-specific expression of TSLP leads to transgene-induced allergic airway inflammation characterized by a massive infiltration of leukocytes, goblet cell hyperplasia, and subepithelial fibrosis, as well as by increased serum IgE levels [7].

TSLP is a typical four-helix-bundle cytokine 140 amino acid residues in length and was first cloned in humans in 2001 [8-10]. The human TSLP gene is localized on chromosome 5q22, interestingly close to the gene cluster encoding several Th2-related cytokines such as IL-4, IL-5, IL-9, and IL-13 [7,11]. Human TSLP is produced by different cell types in atopic asthma, mainly by epithelial and smooth muscle cells and induces an inflammatory Th2 response. The TSLP receptor (TSLPR) is a heterodimeric cytokine receptor consisting of the IL-7 receptor alpha chain (IL-7Rα) and a TSLP-specific receptor chain with similarity to the common gamma receptor chain (γc). The TSLPR, also known as CRLF2, is expressed in heart, skeletal muscle, kidney and liver, but also on asthma-relevant dentritic cells [9,12]. In this review, the signal transduction around human TSLP in the cascade of events in the development of atopic asthma is discussed. We first describe the regulation of TSLP production in airway epithelial and other cells, then cover the TSLPR-mediated effects on TSLP target cells such as DCs and mast cells, and finally treat the DC-triggered onset of a specific Th2 response.

### Regulation of TSLP expression

In the human airway system, fibroblasts, smooth muscle cells, epithelial cells and mast cells all have the potential to produce TSLP [14-18]. Airway epithelial cells (AECs) were found to have increased TSLP mRNA levels in human asthmatics [4]. Importantly, overexpression of TSLP in AECs induces experimental asthma in mice [7].

TSLP expression is enhanced by different stimuli with relevance in asthma. Primary small airway epithelial cells (SAECs) produce biologically active TSLP in response to bacterial peptidoglycan, and lipoteichoic acid as well as to poly I:C (mimicking viral double-stranded RNA) [16]. IL-1β and TNF-α, two cytokines associated with pulmonary inflammation and strongly upregulated in the asthmatic lung [19,20] can, under appropriate conditions, induce human TSLP expression in normal human bronchial epithelial cells (NHBECs) [15,17], SAECs [16] and human airway smooth muscle cells (HASMCs) [18]. Similarly, TGF-β, IFN-β, IL-4, IL-13, and, in particular, a combination of TNF-α and IL-4 or IL-13 upregulate TSLP expression in NHBEs [17].

It is established that rhinovirus and respiratory syncytial virus (RSV) can trigger exacerbations of asthma [21]. TSLP expression in human bronchial epithelial cells is stimulated by both viruses and an involvement of signal transduction through p38 and Jun kinase (JNK) has been demonstrated [22]. Stimulation of TSLP expression evoked by rhinoviral dsRNA and RSV proteins via toll-like receptors (TLRs) is synergistically enhanced by IL-4, indicating a contribution of JAK/STAT signalling [17]. The notion of cooperative signalling to TSLP gene transcription by cytokine and toll-like receptors is supported by the observation that tumor necrosis factor- (TNF-) α and IL-4 or IL-13 jointly drive TSLP expression in NHBECs, but none of the factors has a respective effect on its own. The induction of TSLP by combination of TNF-α and Th2 cytokines but not by the individual cytokines suggests that NFκB and STATs cooperate in transcriptional regulation of the TSLP gene [17].

HASMCs which also act as effector cells in initiating or perpetuating airway inflammation [23,24], respond by TSLP release to stimulation with TNF-α and IL-1β both *in vitro *and *in vivo *[18]. Pharmacological inhibitors of ERK1/2 and p38, but not blockers of phosphatidylinositid-3 kinase (PI-3K) specifically suppress TSLP secretion induced by both factors individually or combination, suggesting that TSLP expression in HASMC is controlled via MAPK pathways [18]. Crosstalk of NFκB- and MAPK pathways is suggested by experiments in cells with mutated mediators NFκB and Ras which show a strong decrease in transcriptional activity of the human TSLP promoter [25].

Studies employing deletion constructs of the TSLP gene promoter indicated that a DNA fragment extending from 3.74 to 3.86 kb upstream of the transcriptional start site contains a cis element required for transcriptional induction by IL-1β. Inspection of this ~120-bp sequence revealed consensus cognate elements for NFκB and IRF-1 as well as a putative AP-1 binding site [15]. Mutations in these motifs indicated that the induction of TSLP gene expression seen in cells stimulated with IL-1β is likely to be mediated through NFκB, whose subunits p65/p50 bind to the NFκB cognate motif in the human TSLP promoter. One of the major pathways for NFκB activation involves the phosphorylation of the inhibitor IκBα, which is followed by IκBα degradation and the subsequent migration of NFκB dimers (each monomer consisting of a p50 and a p65 subunit) from cytoplasm to the nucleus [26]. A dominant-negative mutant of IKKβ (IκB) inhibits IL-1β-mediated transcription of the TSLP gene [15].

Since TLSP induction in the airway epithelium of asthmatics appears to be a associated with allergen contact, it is important to note that engagement of TLRs by allergene provocation activates NFκB [27]. TLR2, TLR3, TLR8, and TLR9 can all induce human TSLP expression in airway epithelial cells [15,17], suggesting that TSLP may become upregulated in the lung epithelium upon allergen challenge. In line with this hypothesis, we have recently observed that direct stimulation of lung epithelial cells with different allergens induces the expression of TSLP mRNA (Borowski et al., manuscript in preparation).

Viral dsRNA is sensed, apart from TLR3, also by the recently identified cytosolic RNA helicases RIG-I and MDA5 [28,29]. Activation of TLR3, RIG-I, and MDA5 by dsRNA is transmitted to transcription factors NFκB and IRF-3, leading to transcriptional upregulation of pro-inflammatory genes and expression of type I interferons including IFN-β. siRNA experiments suggested that TSLP is directly induced by dsRNA in airway epithelial cells, and that the response is mediated by a pathway involving TLR3, NFκB and and IRF-3, but is independent of interferon signalling. Enhancement of dsRNA-dependent TSLP expression by IL-4 is significantly inhibited by siRNA targeting STAT6, supporting the notion of STAT6 as an important transcription factor in the control of TSLP expression [17].

Very recent work showed that TSLP expression in the murine lung is influenced by peptidyl-propyl isomerase (PIN1), an important regulator of survival-promoting and proinflammatory cytokines in T-cells. Active PIN1 inactivates adenosine-uridine binding factor 1 (AUF1), whose function is to destabilize mRNA by interaction with adenosine-uridine rich elements. Since TSLP expression is blocked by a PIN1 inhibitor after challenging lung with allerges and the 3'-untranslated region of TSLP mRNA contain an AUF1 binding site, PIN1 is likely to be a modulator of TSLP expression in asthma at the posttranscriptional level [30].

### Activation of the TSLP receptor and intracellular signal transduction

The specific, low affinity TSLP receptor α chain (TSLPRα) is a member of the hematopoietic (type 1) cytokine receptor family. In combination with the IL-7Rα chain it forms the heterodimeric TSLP receptor (TSLPR) which, upon TSLP binding, transmits signals towards STAT activation and proliferation into the cell interior [8,9,31,32]. The TSLPRα chain has some atypical features for a type 1 cytokine receptor, both in its extracellular and intracellular region. The exodomain, for instance, lacks one of the four cysteine residues conserved within the receptor family, perhaps indicating a unique tertiary structure. Intracellularly, the TSLPRα lacks one of the two conserved sequence boxes present in other cytokine receptors that govern the interaction with Janus kinases (JAKs).

**Figure 1:**
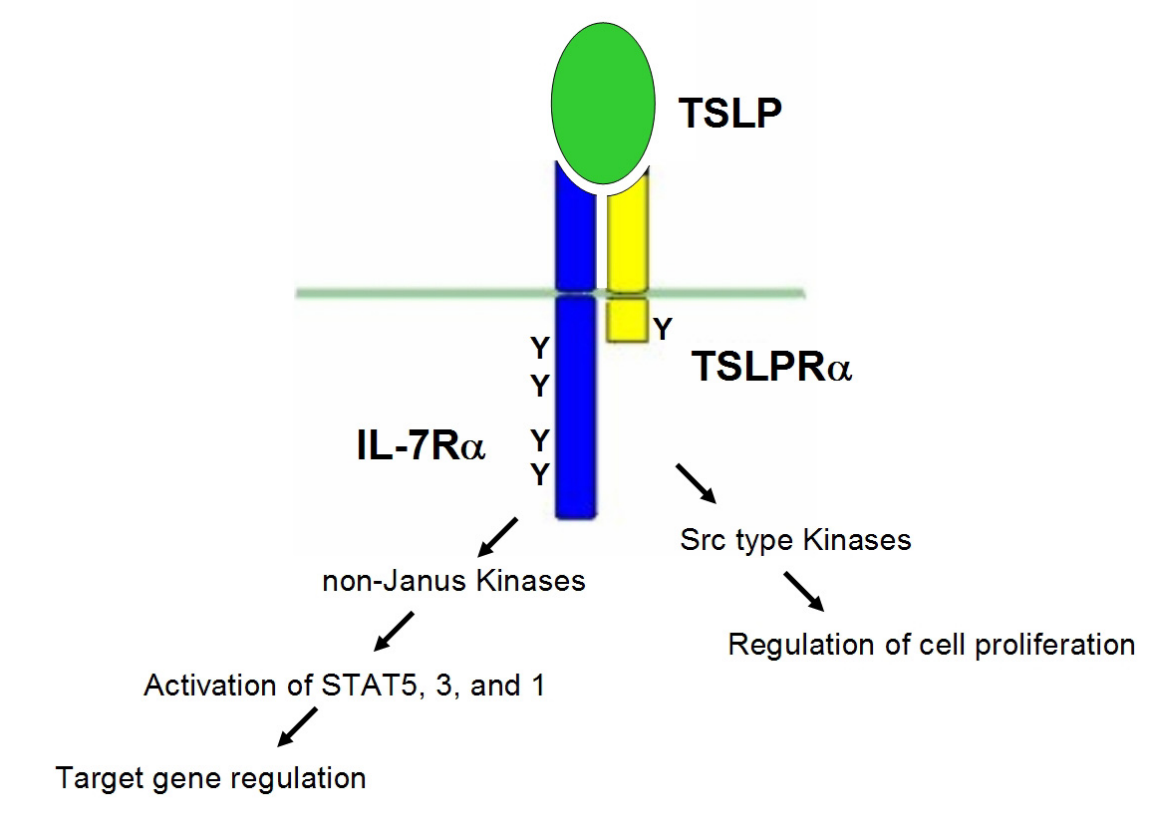
**Structure and signal transduction of the heterodimeric TSLP receptor complex.** For details see text.

Signal transduction emanating from the dimerized TSLPR is similar to signalling from the IL-7R, reflecting the overlapping utilization of the IL-7Rα chain by the two systems. The IL-7 receptor utilizes the γc chain as a dimerization partner for IL-7Rα, which recruits JAK3 via its box1 element. Ligand-induced crosslinking of both the TSLP and IL-7 receptors results in the activation of STAT5 and STAT3 and concomitant specific gene regulation [8,33,34]. However, unlike the IL-7R, the TSLPR appears to drive STAT activity via an uncommon mechanism without an involvement of Janus kinases. Evidence for this interpretation comes from experiments showing that no JAK phosphorylation was evoked by the activated TSLPR and that dominant-negative forms of JAK1 and JAK2 did not block TSLP-mediated STAT5 activation [35]. It was puzzling, however, that fusions of TSLPR cytoplasmic domain with the exodomains of the erythropoietin receptor or CD8 did activate JAK2 upon ligand-dependent homo-dimerization [36-38]. From results obtained with dominant-negative versions of Tec, a role of this cytoplasmic Src-related tyrosine kinase in the TSLPR-mediated activation of STAT5 was inferred [33]. Src-type mediators are also involved in proliferative signaling, TSLP-induced cell proliferation is blocked by the Src family inhibitor PP1 [34]. While both STAT5 activation and cell proliferation require the box1 domain of TSLPRα and IL-7Rα, the single intracellular tyrosine residue of TSLPRα receptor is critical only for proliferative signaling, but not for TSLP-dependent STAT5 activation [34]. Apart from STAT5, TSLP initiates STAT3 phosphorylation in murine DCs without the induction of any of the four Janus kinases (JAKs) [9,31,32] and activates STAT1 in murine pro B cells (A. Wohlmann and K. Friedrich, unpublished results). TSLP does not stimulate the activation of ERK1/2 and p70S6K [8]. Thus, neither the MAPK pathway nor the p70S6K pathway appear to be involved in the signal transduction pathway elicited by TSLP. Details of TSLPR signaling are far from being settled, but the present view is that Src type kinases are mediating the proliferative response and unknown (non-JAK) kinases are critical for STAT activation and, ultimately, regulation of target genes (Figure 1).

### Activation of effector cells of the innate and adaptive immune system through TSLP

**Figure 2:**
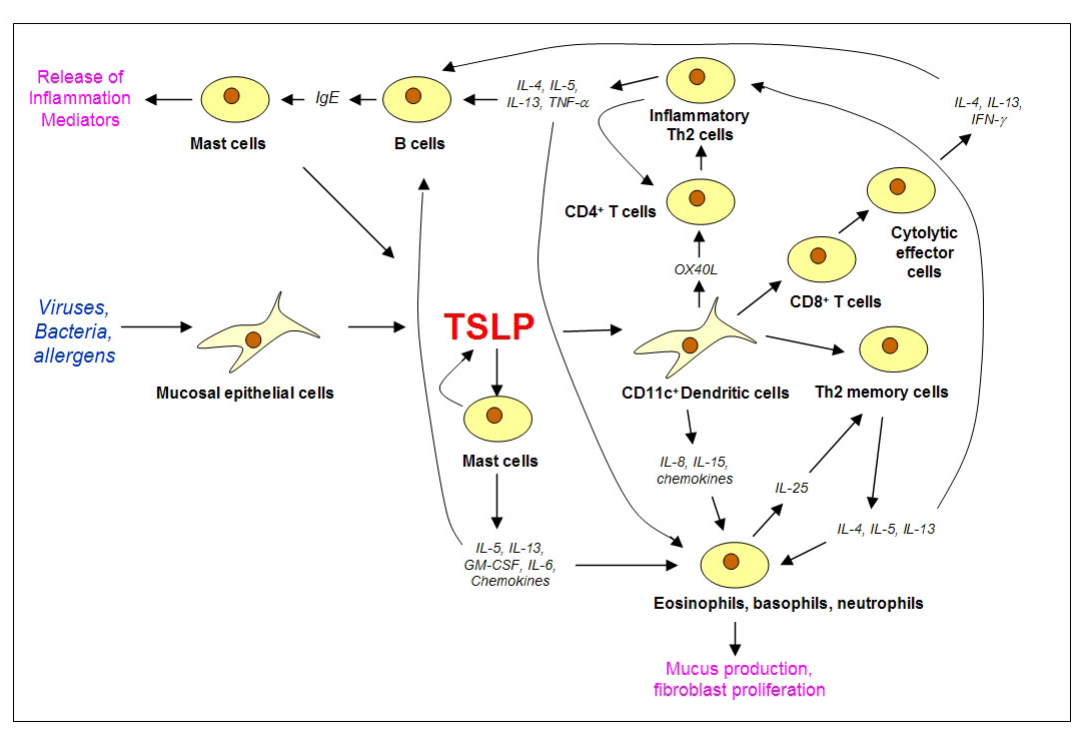
**Central role of TSLP in the orchestration of an asthmatic response upon contact of the airway epithelium with allergens or other challenging agents.** Intercellular communication among different cell types via cytokines evokes activity of the native (bottom part) as well as the adaptive (upper part) of the immune system. For details see text.

Compelling evidence has been acumulated for a determinative role of TSLP in the initiation and maintenance of the allergic response in the context of atopic asthma [5,6]. Human TSLP is able to directly activate effector cells of the innate immune system like mast cells (MCs), which are known to play an important role in the pathogenesis of atopic diseases [39,40]. Functional receptors for TSLP are expressed *in vivo *on MCs infiltrating the bronchial mucosa of asthmatic patients as revealed by immunostaining of biopsy specimen [16]. In inflammatory conditions mimicked by the presence of IL-1 and TNF α, TSLP is a potent activator of MCs leading to the production of high levels of proinflammatory Th2 cytokines and chemokines such as IL-5, IL-13, IL-6, GM-CSF, CXCL8, and CCL1 [16] (Figure 2). Signaling pathways underlying this complex gene regulation have not been characterized yet.

Apart from mast cells, the second main sentinel of the innate immune system is represented by DCs localized at the epithelial surface. DCs are also operative in the creation of a microenvironment that directs T cells towards Th1 or Th2 differentiation. A strong Th2 type response is typical in the context of the allergic syndrome. Human TSLP strongly activates immature CD11c^+ ^DCs while it does not appear to have any direct biological effects on B cells, T cells, NK-cells or neutrophils [9,14,41]. TSLP induces DCs to up-regulate the expression of major histocompatibility class I and II and costimulatory molecules, including CD40, CD80, CD86. Importantly, it also strongly upregulates expression of the mRNA for OX40L, a member of the TNF superfamily that has been implicated in the initiation of Th2 cell responses [42-44]. TSLP also stimulates DCs to produce the Th2-attracting chemokines TARC (thymus and activation regulated chemokine, CCL17) and MDC (macrophage derived chemokine, CCL22) [14], as well as IL-8, IL-15 and eotaxin-2, clearly suggesting that TSLP-activated DCs may represent an initial key step in the development of allergic inflammation [34,45]. In the asthmatic bronchial mucosa, elevated expression of TSLP was also accompanied by and correlated with elevated expression of the CCR4 ligands TARC and MDC at the mRNA level [4]. As revealed by a comparative global transcriptome analysis of naive and TSLP treated DCs, TSLP does not stimulate DCs to produce the Th2-polarizing cytokine IL-4 and, at the same time, suppresses the anti-inflammatory cytokine IL-10 as well as interferon- (IFN-) γ [45].

When TSLP treated DCs are stimulated with CD40 ligand, they induce the differentiation of CD8^+ ^T cells into cytolytic effector cells which produce IFN-γ as well IL-4 and IL-13. Interestingly, expression of these ctokines has as before been considered mutually exclusive [46] (Figure 2).

### Indirect effects of TSLP on Th2 differentiation via OX40L

OX40L, a member of the TNF superfamily has been identified as crucial mediator of Th2 cell responses driven by DCs [43,47]. In mice, blocking of OX40L by inhibitory antibodies inhibited the immune response induced by TSLP, indicated by reduction of cytokine secretion, Th2 inflammatory cell infiltration and IgE production [48]. It appears that OX40L and IL-4 act synergistically and sequentially in driving Th2 cell responses in cocultures of T cell and DCs activated by TSLP [10,45]. Interestingly, in the presence of IL-12, OX40L is unable to induce a Th2 cell response but rather directs T cell differentiation towards the Th1 phenotype, indicating a functional dominance of IL-12 over TSLP [45].

Historically, CD4^+ ^Th2 cells are defined as effector T cells with the capacity to produce IL-4, -5, -10, and -13 [49,50]. IL-4 and IL-13 are typical pro-inflammatory cytokines, but IL-10 does not appear to contribute to allergic inflammation in either humans or mice [51,52], but even suppresses allergic inflammation [53-55]. Importantly, dendritic cells activated by TSLP prime naive CD4^+ ^T cells to differentiate into a particular subtype of Th2 cells that produce the classical Th2 cytokines IL-4, IL-5 and IL-13, but no IL-10. It is also remarkable that these special Th2 cells secrete very high levels of TNF-α [14]. TNF-α is prominent in asthmatic airways and genotypes that correlate with increased TNF-α secretion are associated with an increased risk of asthma [56]. Because of their unique profile of cytokine production, Th2 cells induced by TSLP-activated DCs have been designated inflammatory Th2 cells to discriminate them from the classical regulatory Th2 cells [10] (Figure 2).

Th1 and Th2 cell differentiation is regulated by the key transcription factors T-bet for Th1 and GATA-3 and c-Maf for Th2. Th1 cells express high levels of T-bet but low GATA-3 and c-Maf, while Th2 cells show a reverse expression pattern, hence these transcription factors can be used as molecular markers for Th1 or Th2 cells [57]. CD4^+ ^T cells primed by TSLP-activated DCs show the typical Th2 pattern high GATA-3 and c-Maf and low T-bet. However, IL-12 can override this Th2-specific gene regulation by inhibiting GATA-3 and c-Maf and strongly up-regulating T-bet [Ito *et al. *2005]. Regulation processes behind this phenomenon may involve temporal IL-12-induced upregulation of the IL-12R signaling subunit (IL-12Rβ2) and concomitant signal transduction via STAT4, which has been observed in CD4+ cells upon activation of OX40 [58].

TSLP has also been discussed as a player in the maintenance and regulation of Th2 memory cells. This interpretation comes from the finding that DCs activated by TSLP can induce an expansion of a CD4^+ ^T cell subset expressing the prostaglandin D2 receptor (CRTH2), a property of human Th2 central memory T cells [59]. Interestingly, TSLP-activated DCs enhance the allergy-inducing properties of Th2 memory cells by up-regulating their expression of pro-allergic genes, particularly IL-17RB, the receptor for IL-25. IL-25, in turn, was shown to trigger the proliferation of Th2 memory cells and increase to the production of IL-4, IL-5, and IL-13, but not TNF-α or IFN-γ. These results suggest that IL-25 may costimulate the proliferation and further polarization of Th2 memory cells induced by TSLP-activated DCs [60].

Importantly for the development of asthmatic symptoms, the activation of inflammatory Th2 cells through TSLP and their production of the inflammatory cytokines IL-4, IL-5, IL-13 and TNF-α triggers IgE production, eosinophilia, mucus production and fibroblast proliferation [61,62]. The effector mechanisms of atopic asthma ultimately involve IgE-coated mast cells that undergo degranulation upon contact with the allergen and induce an immediate response, leading to the symptoms of local inflammation and bronchospasm [63].

## Conclusion

In atopic asthma, many different agents such as viruses, bacteria and allergens can induce a TSLP-dependent inflammatory response, leading to an inappropriate activation of both the innate and the adaptive immune system. With regard to the innate branch of the response, TSLP acts on mast cells and dendritic cells as well as, according to recent results, natural killer cells. Mast cells play a prominent role in the development of asthmatic symptoms, because they secrete inflammatory cytokines in response to TSLP. The fact that mast cells also produce TSLP indicates a potential amplification loop by the action of mast cell-derived TSLP on epithelial DCs. A critical switch governed by DCs is the TSLP-induced expression of OX40L, a Th2 cell polarizing signalling molecule. A further emerging role of TSLP is the generation of Th2 memory T cells. The IL-25-mediated collaborative interactions between eosinophils/basophils and Th2 memory cells perhaps propagate a positive feedback loop between innate effector and adaptive immunity, leading to the amplification of allergic inflammation (Figure 2).

## List of abbreviations used

AEC: Airway Epithelial Cells; DCs: Dendritic Cells; GM-CSF: Granulocyte-Macrophage Colony Stimulating Factor; HASMCs: Human Airway Smooth Muscle Cells; IgE: Immunoglobulin E; IL: Interleukin; IFN: Interferon; JAK: Janus Kinase; JNK: Jun Kinase; MAPK: Mitogen Activated Protein Kinase; MCs: Mast Cells; NFκB: Nuclear Factor κB; NHBECs: Normal Human Bronchial Epithelial Cells; NK cells: Natural Killer Cells; PI-3K: Phosphatidylinositid 3-Kinase; SAECs: Small Airway Epithelial Cells; TLR: Toll-like Receptor; TNF: Tumor Necrosis Factor; TSLP: Thymic Stromal Lymphopoietin; TSLPR: Thymic Stromal Lymphopoietin Receptor; STAT: Signal Transducer and Activator of Transcription.

## Competing interests

The authors declare that they have no competing interests.

## Authors' contributions

KS and AB collected information from the literature and prepared iniatial versions of large parts of the manuscript. MK provided additional information from an immunological and clinical point of view and edited major parts of the manuscript. KHF conceived the overall organization of the manuscript, added extended sections of text and did the final editing. All authors read and approved the final manuscript.
